# An Improved Segformer for Semantic Segmentation of UAV-Based Mine Restoration Scenes

**DOI:** 10.3390/s25123827

**Published:** 2025-06-19

**Authors:** Feng Wang, Lizhuo Zhang, Tao Jiang, Zhuqi Li, Wangyu Wu, Yingchun Kuang

**Affiliations:** 1 College of Information and Intelligence, Hunan Agricultural University, Changsha 410128, China; wangfeng_0825@stu.hunau.edu.cn (F.W.); zhanglizhuo@hunau.edu.cn (L.Z.); 2 Technology Innovation Center for Ecological Conservation and Restoration in Dongting Lake Basin, Ministry of Natural Resources, Changsha 410004, China; hunanjhzhb@163.com; 3 Hunan Center of Natural Resources Affairs, Changsha 410004, China; 4 School of Computer and Control Engineering, Northeast Forestry University, Harbin 150006, China; 3056615581@nefu.edu.cn; 5 School of Computer Science, University of Liverpool, Liverpool L69 3DR, UK; wangyu.wu@liverpool.ac.uk

**Keywords:** semantic segmentation, mine restoration, UAV image, Segformer, attention mechanism, feature fusion

## Abstract

Mine ecological restoration is a critical process for promoting the sustainable development of resource-dependent regions, yet existing monitoring methods remain limited in accuracy and adaptability. To address challenges such as small-object recognition, insufficient multi-scale feature fusion, and blurred boundaries in UAV-based remote sensing imagery, this paper proposes an enhanced semantic segmentation model based on Segformer. Specifically, a multi-scale feature-enhanced feature pyramid network (MSFE-FPN) is introduced between the encoder and decoder to strengthen cross-level feature interaction. Additionally, a selective feature aggregation pyramid pooling module (SFA-PPM) is integrated into the deepest feature layer to improve global semantic perception, while an efficient local attention (ELA) module is embedded into lateral connections to enhance sensitivity to edge structures and small-scale targets. A high-resolution UAV image dataset, named the HUNAN Mine UAV Dataset (HNMUD), is constructed to evaluate model performance, and further validation is conducted on the public Aeroscapes dataset. Experimental results demonstrated that the proposed method exhibited strong performance in terms of segmentation accuracy and generalization ability, effectively supporting the image analysis needs of mine restoration scenes.

## 1. Introduction

China is endowed with abundant mineral resources and has a long history of mining activities [1,2]. High-quality mineral resources have long played an irreplaceable role in national economic development. However, prolonged and intensive mining has caused severe ecological degradation, leading to a range of environmental issues, such as land desertification, soil erosion, and vegetation loss, which in turn pose serious threats to the stability and sustainability of regional ecosystems [3,4]. With the growing emphasis on ecological civilization and environmental protection, mine restoration and ecological reconstruction have become critical components of contemporary ecological governance [4,5]. There is an urgent need to employ scientific and efficient technological approaches to improve the ecological environment of mining areas and promote the rational utilization of land resources.

Traditional methods for monitoring the effectiveness of mine restoration primarily rely on field surveys or remote sensing image analysis. However, these approaches are typically labor-intensive, time-consuming, and prone to subjective bias, with limited accuracy—falling short of the high-efficiency, high-precision, and automated analysis requirements of modern mine rehabilitation practices [6,7]. In recent years, low-altitude unmanned aerial vehicle (UAV) remote sensing has emerged as a promising tool for ecological monitoring in mining areas due to its flexibility in image acquisition, high spatial resolution, and short revisit cycles. Compared to traditional satellite remote sensing, UAV platforms are better suited to capture fine-grained local features in mine restoration zones [8,9]. However, UAV imagery alone cannot directly reveal the status of land cover restoration—it must be combined with advanced image interpretation algorithms for in-depth semantic understanding.

With the rapid advancement of deep learning, computer vision has achieved notable success in object recognition and environmental monitoring [10]. Semantic segmentation, as a core task in image understanding, performs pixel-level classification of different land cover types within an image. It provides essential technical support for evaluating ecological restoration and land-use planning. Compared to natural images, UAV-acquired imagery often exhibits large top-down viewing angles, significant variations in object scale, and ambiguous class boundaries. These challenges are particularly pronounced in mine restoration scenes, which are characterized by a high proportion of small objects and complex spatial structures, thereby increasing the difficulty of semantic segmentation.

In the development of semantic segmentation models, convolutional neural networks (CNNs) have demonstrated strong local feature extraction capabilities and performed well in early segmentation tasks. Classic models, such as FCN [11], U-Net [12], and the DeepLab series [13,14,15], have been widely adopted in remote sensing image analysis. However, CNNs inherently struggle to capture long-range dependencies and model global context, which can result in the omission of small targets and blurred object boundaries. The introduction of the Vision Transformer (ViT) [16] has brought transformative progress to visual tasks by offering superior global modeling capabilities—particularly suitable for handling the complex spatial structures and scale variations present in remote sensing imagery [17]. Nevertheless, pure Transformer models often suffer from large parameter counts and high computational costs. For instance, models like ViT and SETR [18], while powerful, are limited in practical remote sensing applications due to their computational demands.

Segformer [19], a lightweight Transformer-based semantic segmentation network, balances global modeling capability with computational efficiency. It employs the Mix Vision Transformer (MiT) as the encoder, which features a strong multi-scale representation ability and can construct semantic structures without positional encoding. Its decoder is designed with a simple architecture and offers fast inference, making it well-suited for deployment in complex remote sensing scenarios. Given these advantages, this study adopts Segformer as the baseline architecture and introduces targeted enhancements to better accommodate the requirements of mine restoration scenarios—particularly in improving the segmentation of multi-scale objects, boundary regions, and small targets.

In semantic segmentation tasks, shallow features extracted by the backbone network preserve rich spatial details, while deeper features capture more abstract semantic representations. Segformer concatenates and fuses features from multiple scales after alignment through multilayer perceptron (MLP), which enables basic feature modeling. However, this design remains limited in preserving spatial detail, facilitating semantic interaction, and detecting small targets. To better adapt to the characteristics of remote sensing images in mine restoration scenes, we propose an improved architecture based on Segformer. Specifically, we introduce a multi-scale feature-enhanced feature pyramid network (MSFE-FPN) between the encoder and decoder. This module strengthens cross-scale interactions through a top-down pathway and lateral connections, enhancing multi-scale feature representation. In the deepest lateral connection, we integrate the selective feature aggregation pyramid pooling module (SFA-PPM), which utilizes multi-scale pooling and channel-wise selection mechanisms to improve global perception and long-range dependency modeling. Additionally, we incorporate the efficient local attention (ELA) module [20] in the remaining lateral connections to enhance edge feature learning and small-object modeling using lightweight attention mechanisms. These improvements collectively enhance the model’s robustness and segmentation accuracy in complex mine restoration scenarios. The main contributions of this paper are summarized as follows:

(1) We propose a Segformer-based semantic segmentation model with an encoder–FPN–decoder architecture. An FPN is introduced between the encoder and decoder to strengthen top-down fusion of multi-level features, addressing the limitations of the original Segformer in semantic–spatial information interaction.
(2) We design the multi-scale feature-enhanced FPN (MSFE-FPN). Specifically, we incorporate a selective feature aggregation pyramid pooling module (SFA-PPM) in the deepest lateral connection to enhance global semantic perception and integrate efficient local attention (ELA) modules into other lateral connections to improve edge feature extraction and small-object recognition.
(3) We construct a UAV-based semantic segmentation dataset for mine restoration—the HUNAN Mine UAV Dataset (HNMUD), which contains 2700 high-resolution annotated images. Additionally, we conduct generalization experiments on the publicly available Aeroscapes dataset to validate the cross-domain adaptability and robustness of the proposed model.

## 2. Related Work

### 2.1. Deep Learning for Image Segmentation

Chen et al. [11] pioneered the fully convolutional network (FCN), replacing traditional fully connected layers with convolutional layers to enable end-to-end pixel-level prediction, marking the beginning of deep learning applications in semantic segmentation. Chen et al. [13,14,15] introduced the DeepLab series, which utilized dilated convolutions and fully connected conditional random fields (CRFs) to capture multi-scale contextual information and refine object boundaries. Ronneberger et al. [12] proposed U-Net, an encoder–decoder architecture with skip connections originally designed for biomedical image segmentation, which has since become widely adopted in general semantic segmentation tasks. Badrinarayanan et al. [21] presented SegNet, which also employed an encoder–decoder structure and used pooling indices during upsampling to strike a balance between segmentation accuracy and computational efficiency. Zhao et al. [22] proposed PSPNet, which introduced a pyramid pooling module to aggregate contextual information at multiple scales, enhancing the model’s ability to handle complex scenes. With the development of attention mechanisms, the Transformer architecture has been gradually adopted for vision tasks. Vaswani et al. [17] first introduced the Transformer, a model based purely on self-attention mechanisms, laying the theoretical foundation for visual Transformers. Building on this, Dosovitskiy et al. [16] proposed the Vision Transformer (ViT), which splits images into fixed-size patches and treats them as sequences, achieving performance comparable to or surpassing CNNs on large-scale datasets. Liu et al. [23] introduced the Swin Transformer, which employs a hierarchical structure and shifted windows to reduce computational complexity, achieving state-of-the-art results in classification, detection, and segmentation. The Swin Transformer [23], a hierarchical vision transformer with shifted windows, significantly improved performance on dense prediction tasks, such as semantic segmentation, by enabling efficient computation and modeling of long-range dependencies. Zheng et al. [18] proposed SETR, the first model to apply a pure Transformer architecture to semantic segmentation, offering enhanced long-range dependency modeling capabilities. Xie et al. [19] developed Segformer, which combines a hierarchical Transformer encoder with a lightweight MLP decoder, achieving superior segmentation accuracy with efficient computation. He et al. [24] proposed Masked Autoencoders (MAE), a self-supervised pretraining method that reconstructs masked image patches, enabling robust global feature learning.

### 2.2. Semantic Segmentation of UAV Images

In recent years, deep neural networks have drawn increasing attention in the field of semantic segmentation based on UAV images, owing to their powerful feature representation capabilities. Giang et al. [25] proposed a U-Net-based model for high-resolution image segmentation, achieving accurate classification of surface cover types in mining areas—such as vegetation and bare land—with excellent performance in edge refinement and small-object detection. Saxena et al. [26] developed a lightweight segmentation network incorporating attention mechanisms for small fire detection, showing high sensitivity in identifying small-scale fire regions and offering potential for early wildfire monitoring. Hanyu et al. [27] introduced AerialFormer, which combines multi-scale feature fusion and the global modeling capabilities of Transformers, achieving state-of-the-art performance on several remote sensing datasets. Wang et al. [28] proposed UnetFormer, integrating the traditional U-Net structure with Transformer modules to enhance representation learning in urban remote sensing scenes while maintaining efficiency. He et al. [29] designed a deep neural network with uncertainty-aware mechanisms for building extraction from remote sensing imagery, achieving robust segmentation under occlusion and complex backgrounds by modeling predictive uncertainty. Ghali et al. [30] developed a CNN–Transformer hybrid framework for wildfire detection and segmentation, which integrates spatial attention and multi-scale feature fusion to improve performance in complex fire scenes. Cao et al. [31] focused on unstructured rural road extraction, proposing a semantic segmentation network with enhanced spatial pyramid pooling and multi-scale fusion modules, significantly improving edge recognition accuracy. Chen et al. [32] presented an improved DeepLabv3+ model that integrates ResNet101 and an Efficient Channel Attention (ECA) mechanism for landslide area identification, effectively enhancing channel-wise feature responses. Xu et al. [33] proposed an improved U-Net architecture for precise single-tree segmentation, supporting fine-grained forest management and monitoring. Zheng et al. [34] explored lightweight segmentation networks for crop classification in UAV imagery, striking a balance between segmentation accuracy and real-time processing demands. Jin et al. [35] proposed EMR-HRNet, a novel multi-scale feature fusion network tailored for landslide segmentation tasks. Wang et al. [36] proposed an improved Segformer model for the extraction of winter wheat planting areas with complex structures in multispectral remote sensing images.

## 3. Materials and Methods

### 3.1. Datasets

#### 3.1.1. HUNAN Mine UAV Datasets

To support semantic segmentation research in mine ecological restoration, this paper constructs a representative UAV-based remote sensing dataset, named the HUNAN Mine UAV Dataset (HNMUD). The dataset was collected using DJI drones across typical mining areas in 14 prefecture-level cities of Hunan Province, China.

**Figure 1:**
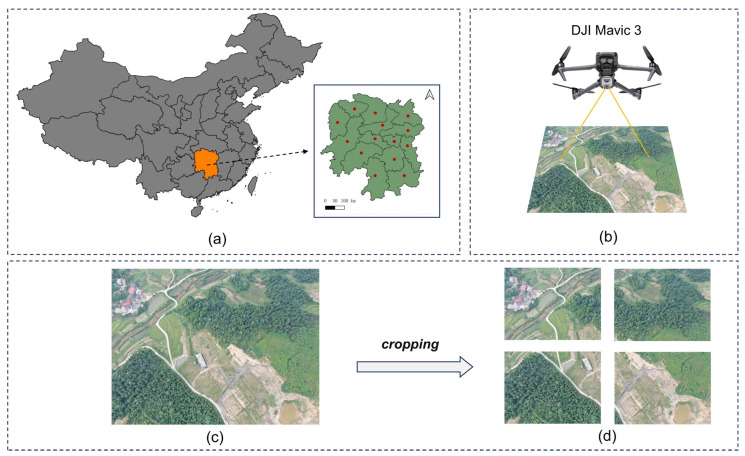
(**a**) Geographic distribution of UAV data acquisition sites across 14 prefecture-level cities in Hunan Province. (**b**) Illustration of the DJI Mavic 3 used for aerial photography over typical mining areas. The original aerial image (**c**) is divided into smaller patches of size 1024 × 1024 pixels (**d**) through a cropping operation.

As shown in Figure 1a, the red dots mark the locations of the surveyed mines, covering diverse terrain types, vegetation coverage levels, and various stages of ecological restoration. During data acquisition, the DJI Mavic 3 UAV was employed (Figure 1b) to perform both nadir and oblique aerial photography, ensuring rich multi-angle scene information. This process resulted in the collection of 1500 wide-view raw images under various environmental and lighting conditions. Figure 1c shows an example of an original wide-view image, while Figure 1d demonstrates the image cropping process, where the original image was divided into several smaller images, each sized 1024 × 1024 pixels, to facilitate detailed analysis. Subsequently, low-quality or redundant images were filtered out, and data augmentation techniques, such as rotation, horizontal flipping, and brightness adjustment, were applied. The final dataset contained 2700 high-resolution images for training and evaluation. Each image in the dataset was annotated at the pixel level using the Labelme tool to ensure accuracy and consistency in semantic labeling. The dataset included six semantic categories: background, vegetation, farmland, building, ground, and mine.

#### 3.1.2. Aeroscapes Dataset

The Aeroscapes dataset is a high-resolution remote sensing image collection specifically designed for semantic segmentation tasks in the context of aerial and UAV-based perspectives [37]. It comprises 3269 RGB images with a resolution of 1920 × 1080, extracted from 141 video sequences captured by UAVs operating across diverse urban and rural environments. The dataset provides pixel-level annotations for 12 semantic categories, including ‘background’, ‘person’, ‘bike’, ‘car’, ‘drone’, ‘boat’, ‘animal’, ‘obstacle’, ‘construction’, ‘vegetation’, ‘road’, and ‘sky’—all of which are commonly encountered in aerial imagery. To enhance the generalization capability and robustness of segmentation models, the Aeroscapes dataset incorporates variations in altitude, viewing angles, lighting conditions, and diverse terrain types. Its high-quality pixel annotations and rich scene diversity make it a valuable benchmark for evaluating the performance of semantic segmentation algorithms. In this study, the Aeroscapes dataset was utilized to assess the generalization ability of the proposed model. Given the notable differences in acquisition environments and scene distributions between Aeroscapes and our self-constructed HNMUD, this evaluation setting provided a more rigorous test of the model’s transferability across different UAV-based scenarios.

### 3.2. Methods

#### 3.2.1. The Segformer Network

**Figure 2:**
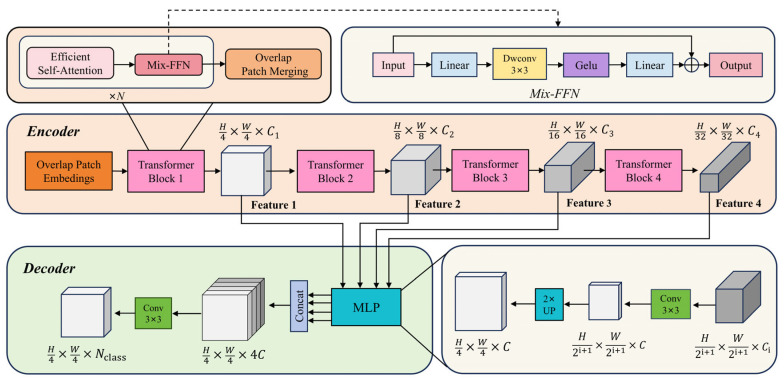
The structure of the Segformer network.

As illustrated in Figure 2, the Segformer architecture is composed of two main components: the encoder and the decoder [19].

The encoder adopts a hierarchical structure, consisting of four stages (Stages 1–4), with each stage corresponding to a Transformer block designed to capture multi-scale semantic features at different spatial resolutions. Each Transformer block is comprised of three core modules: overlap patch embedding (OPE), efficient multi-head self-attention (EMSA), and the mix feed-forward network (Mix-FFN). Specifically, the OPE module divides the input image into overlapping patches and applies convolutional operations to extract patch tokens. Given an input image of resolution H×W×3, a sequence of patch-merging operations across stages produces hierarchical feature maps $F_{i}$, with a resolution of $\frac{H}{2^{i+1}}\times \frac{W}{2^{i+1}}\times C_{i}$, where i ∈{1,2,3,4} and $C_{i+1}>C_{i}$. This design enables efficient downsampling while preserving spatial continuity and edge information, which facilitates the modeling of strong local semantic relationships. Subsequently, the feature maps are passed into the EMSA module. This module first flattens the spatial dimensions to construct a one-dimensional token sequence, then applies an optimized multi-head self-attention mechanism to model long-range dependencies. This allows the network to capture global contextual information effectively while maintaining low computational complexity. To address the limited spatial awareness inherent in traditional Transformers due to the absence of explicit positional encoding, Segformer incorporates a 3×3 depthwise separable convolution within the Mix-FFN module. This convolution is embedded between two linear layers and is responsible for integrating spatial structural information. By eliminating the need for explicit positional encoding—which can degrade performance under varying input resolutions—this design significantly enhances the model’s generalization and robustness in multi-scale scenarios.

Finally, the multi-scale features extracted from the four encoder stages are forwarded to the decoder. The decoder adopts a lightweight MLP-based design, where the feature maps from each stage are first upsampled to a unified resolution of $\frac{H}{4}\times \frac{W}{4}\times C$, then concatenated. A 3×3 convolution is applied to fuse these features, followed by a 1×1 convolution to generate the final semantic segmentation map. This decoder structure not only simplifies the overall network design but also achieves outstanding segmentation performance, particularly when dealing with complex object structures and fine-grained edge details.

#### 3.2.2. The Overview of Improved Segformer Architecture

**Figure 3:**
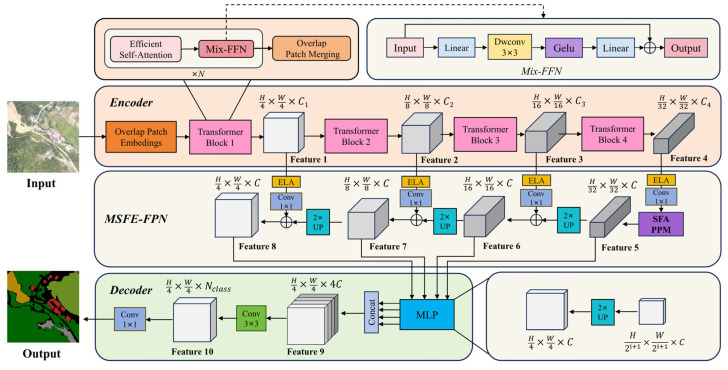
The structure of the improved Segformer model.

Considering the similar spectral characteristics of vegetation, the complexity and diversity of land types in mining areas, and the small scale of buildings, we propose an enhanced semantic segmentation model tailored for UAV-based mine restoration scenes. As illustrated in Figure 3, a novel multi-scale feature enhancement feature pyramid network (MSFE-FPN) was integrated between the encoder and decoder of Segformer to improve feature representation and segmentation accuracy.

This module incorporates two key components: efficient local attention (ELA) and the selective feature aggregation pyramid pooling module (SFA-PPM), which collectively enhance the model’s ability to perceive objects at multiple scales and improve its understanding of complex semantic structures. Specifically, MSFE-FPN first integrates multi-scale features from different encoder stages through lateral connections and then applies a top-down semantic enhancement strategy. During lateral fusion, the ELA module is embedded to reinforce interactions among local contextual features. Additionally, in the enhancement path of the deepest feature map $F_{4}$, the proposed SFA-PPM is employed to improve global semantic modeling and the recognition of large-scale objects. The enhanced multi-scale features generated by MSFE-FPN are subsequently passed to the decoder for final semantic prediction. This architecture not only inherits Segformer’s strength in efficient semantic modeling but also significantly improves the model’s capacity to handle complex structures in mining area images, such as blurred edges, fragmented regions, and occlusions. The design principles and core components of the proposed MSFE-FPN will be elaborated in Section 3.2.3.

#### 3.2.3. The Multi-Scale Feature Enhancement Feature Pyramid Network Structure

**Figure 4:**
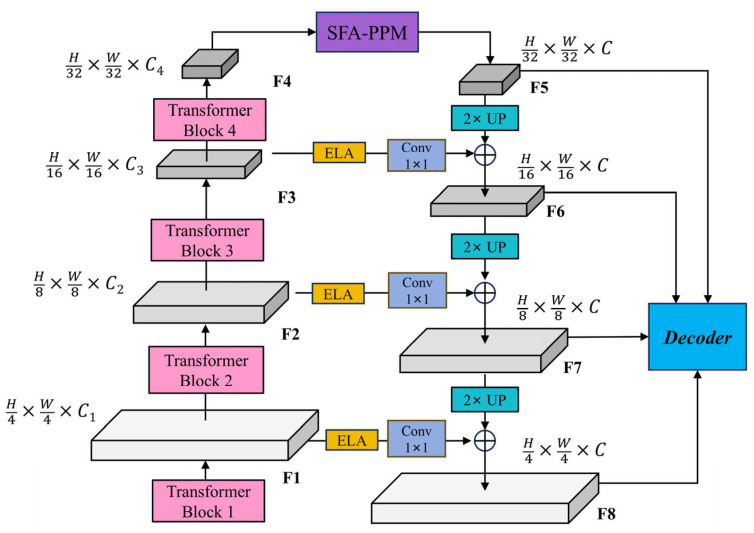
The structure of the MSFE-FPN module.

As aforementioned, to further enhance the feature representation capability of the Segformer architecture, this paper proposes a multi-scale feature enhancement feature pyramid network (MSFE-FPN), which, inspired by the feature pyramid network (FPN) [38], adopts a top-down structure to progressively enhance and fuse multi-level features extracted by the backbone network. While ELA and PPM have been independently validated in prior works, their joint utilization within a unified feature pyramid architecture is non-trivial. In our design, ELA enhances early-stage lateral features where spatial details are rich, whereas SFA-PPM complements this by enriching the deepest feature map with context-aware information. This hierarchical synergy is particularly beneficial for complex UAV mining scenes, where both low-level boundaries and high-level object semantics are crucial for accurate segmentation. The structure is illustrated in Figure 4.

Specifically, the four feature maps extracted by the backbone are denoted as $F_{i}$ with a resolution of $\frac{H}{2^{i+1}}\times \frac{W}{2^{i+1}}\times C_{i}$, where i ∈{1,2,3,4}. MSFE-FPN mainly leverages two mechanisms for feature enhancement and fusion: (1) the selective feature aggregation pyramid pooling module (SFA-PPM) and (2) the efficient local attention (ELA) module.

The deepest feature map $F_{4}$, which contains rich semantic information, is first passed through the SFA-PPM module to perform multi-scale context modeling, resulting in the enhanced feature $F_{5}$. In the top-down pathway, the enhanced feature ${\hat{F}}_{4}$ is progressively upsampled and fused with the shallower feature maps $F_{3}$, $F_{2}$, and $F_{1}$. To preserve detailed spatial information, each shallow feature is first enhanced by the ELA module and then passed through a 1×1 convolution to unify the channel dimension to C, denoted as:

(1) $$L_{i}={Conv}_{1\times 1}(ELA(F_{i})),i\in \{1,2,3\}.$$

The top-down fusion is then performed as follows:

(2) $$F_{5+i}=Up\left(F_{4+i}\right)+L_{4-i},i\in \{1,2,3\},$$

where Up represents 2× bilinear interpolation upsampling. Finally, the enhanced multi-scale feature maps are obtained $\left\{F_{5},F_{6},F_{7},F_{8}\right\}$, which are then fed into the decoder for final semantic segmentation prediction.

#### 3.2.4. The Selective Feature Aggregation Pyramid Pooling Module

**Figure 5:**
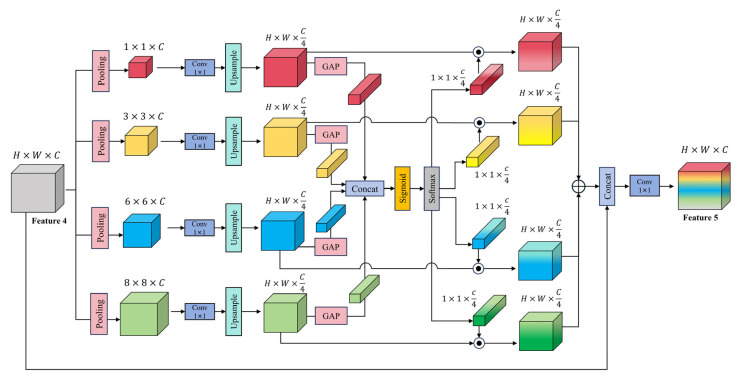
The structure of the SFA-PPM module.

Considering that the deep feature maps extracted by the backbone contain richer semantic information, we propose an enhanced module, named the selective feature aggregation pyramid pooling module (SFA-PPM), as illustrated in Figure 5, to further improve the ability of semantic segmentation models to capture multi-scale contextual dependencies. Based on the original pyramid pooling module (PPM) in PSPNet [22], SFA-PPM integrates a multi-scale selective fusion (MSF) mechanism to adaptively weight and fuse multi-scale features, thereby enhancing multi-scale feature representation.

Specifically, given the final feature map $X\in R^{B\times C\times H\times W}$ extracted from the backbone, SFA-PPM applies average pooling operations at four different spatial scales: 1×1, 3×3, 6×6, and 8×8, resulting in four context feature maps $\left\{y_{0},y_{1},y_{2},y_{3}\right\}$ that encode global-to-local information. These feature maps are then upsampled via bilinear interpolation to match the original resolution and unified to a shape of $H\times W\times \frac{C}{4}$ before fusion.

To achieve adaptive multi-scale fusion, global average pooling (GAP) is first applied to each branch to extract channel-wise context descriptors. These are passed through separate 1×1 convolution layers to compute attention weights:

(3) $$w_{i}={Conv}_{1\times 1}\left(GAP\left(y_{i}\right)\right),i=0,1,2,3.$$

The resulting attention descriptors are concatenated along the scale dimension to form a unified attention tensor:

(4) $$W=Concat\left(w_{0},w_{1},w_{2},w_{3}\right).$$

This tensor is activated with a Sigmoid function and then normalized along the scale dimension using a Softmax function, ensuring that the weights sum to 1 for each channel.

The normalized weights are then split into four separate attention vectors ${\tilde{W}}_{i}$, where i∈{1,2,3,4}, which are used to perform channel-wise weighted fusion of the multi-scale features:

(5) $$X_{att}=\sum_{i=0}^{3}{\tilde{W}}_{i}\cdot y_{i}.$$

Finally, the fused attention-enhanced feature map $X_{att}$ is concatenated to the original feature map X, followed by a 1×1 convolution for dimensionality reduction to obtain the final multi-scale contextual feature:

(6) $$Z=Conv_{1\times 1}\left(Concat\left(X_{att},X\right)\right).$$

#### 3.2.5. The Efficient Local Attention Module

In semantic segmentation tasks, conventional global self-attention mechanisms are effective in modeling long-range dependencies but often incur substantial computational costs when handling high-resolution images. Additionally, they tend to be less responsive to fine-grained details, such as edges, textures, and local patterns. To overcome these limitations, numerous attention mechanisms have been proposed to enhance local feature representation. SENet [39] introduces channel-wise attention by recalibrating features through global average pooling and fully connected layers, thereby emphasizing informative channels. CBAM [40] extracts spatial attention, though fails to model the long-range dependencies crucial for vision tasks, while also reducing the channel dimension of the input feature map. Coordinate attention [41] extends this by embedding positional information into the attention process, improving the localization capability. ECA [42] simplifies the attention computation by eliminating dimensionality reduction and adopting lightweight 1D convolutions to capture local cross-channel interactions. Inspired by these advances, this study integrates the efficient local attention (ELA) [20] module into the proposed MSFE-FPN framework to improve the modeling of local spatial structures while maintaining computational efficiency.

**Figure 6:**
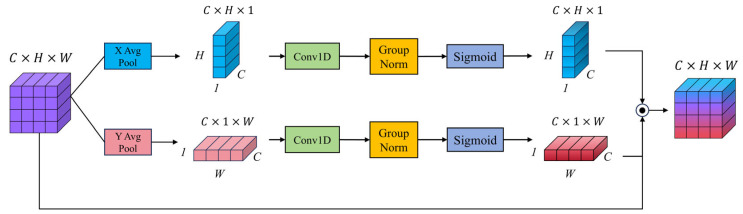
The structure of the ELA module.

As illustrated in Figure 6, given the input feature map $F\in R^{B\times C\times H\times W}$, the ELA module first performs global average pooling along spatial dimensions to obtain compressed features along the width and height directions:

(7) $$F_{h}\left(c,h\right)=\frac{1}{W}\sum_{w=1}^{W}F\left(c,h,w\right),$$

(8) $$F_{w}\left(c,w\right)=\frac{1}{H}\sum_{h=1}^{H}F\left(c,h,w\right).$$

Next, one-dimensional convolution, group normalization, and Sigmoid activation are applied to $F_{h}$ and $F_{w}$:

(9) $$F_{h}^{\prime }=\sigma \left(GN\left(Conv1D\left(F_{h}\right)\right)\right),$$

(10) $$F_{w}^{\prime }=\sigma \left(GN\left(Conv1D\left(F_{w}\right)\right)\right),$$

where σ denotes the Sigmoid activation function and GN denotes group normalization. The resulting attention maps are then broadcasted to the spatial dimensions of the input feature map and element-wise multiplied with the original input, yielding the final output, $F_{out}$, as follows:

(11) $$F_{out}=F\cdot F_{h}^{\prime }\cdot F_{w}^{\prime }.$$

## 4. Experimental Results and Analyses

### 4.1. Experimental Setup and Evaluation Metrics

This paper employed the improved Segformer model with the proposed MSFE-FPN architecture for the semantic segmentation task. During training, each image was resized to 1024 × 1024, and data augmentation techniques, such as random horizontal flipping (with a probability of 50%), random resizing (scale ratio range of 0.5 to 2.0), random cropping (512 × 512), and photometric distortion, were applied, and we randomly split the images into training and test sets in a 8:2 ratio. The model was trained using the Adam optimizer with an initial learning rate of 6 × 10^−5^. A total of 200 training epochs were conducted with a batch size of 8. Additionally, we utilized the ADE20K pretrained weights of the Segformer model to initialize the encoder, which facilitated faster convergence and improved feature extraction performance. During training, the cross-entropy loss function was employed to optimize the model parameters. The specific experimental running environment is detailed in Table 1.

To comprehensively evaluate the performance of the proposed model and baseline methods, four commonly used metrics were adopted: mean intersection over union (*mIoU*), mean pixel accuracy (*mPA*), mean F1-score (*mF1*), and mean recall (*mRecall*). These metrics were calculated across all semantic categories to ensure a balanced and rigorous evaluation of segmentation performance on UAV-based mine restoration scenes.

The *mIoU* evaluates the average overlap between the predicted segmentation and the ground truth across all classes. For class i, the *IoU* is defined as:

(12) $${IoU}_{i}=\frac{{TP}_{i}}{{TP}_{i}+{FP}_{i}+{FN}_{i}},$$

where $TP_{i}$, $FP_{i}$, and $FN_{i}$ denote the number of true positive, false positive, and false negative pixels for class i, respectively. The mean *IoU* over N classes is given by:

(13) $$mIoU=\frac{1}{N}\sum_{i=1}^{N}{IoU}_{i}.$$

The mean pixel accuracy (*mPA*) measures the proportion of correctly predicted pixels for each class and then averages them across all classes:

(14) $$mPA=\frac{1}{N}\sum_{i=1}^{N}\frac{TP_{i}}{{TP}_{i}+{FP}_{i}}.$$

The mean recall (*mRecall*) reflects the model’s ability to correctly identify relevant pixels for each class:

(15) $$mRecall=\frac{1}{N}\sum_{i=1}^{N}\frac{TP_{i}}{TP_{i}+{FN}_{i}}.$$

The mean F1-score (*mF*1) is the harmonic mean of precision and recall for each class, providing a balanced measure between the two:

(16) $$mF1=\frac{1}{N}\sum_{i=1}^{N}\frac{2TP_{i}}{2TP_{i}+{FP}_{i}+{FN}_{i}}$$

### 4.2. Comparison with Other Methods

#### 4.2.1. Experiments on the HUNAN Mine UAV Datasets

We evaluated the improved Segformer model on the UAV-based HNMUD against nine representative segmentation models. As shown in Table 2, U-Net achieved the lowest mIoU (62.43%), while DeepLabv3+ reached 76.20%. Among Transformer-based models, Mask2Former achieved the best results (mIoU 84.32%), followed by Swin Transformer (83.78%) and SETR (76.59%). MSFCA and EMR-HRNet showed stronger performance, with MSFCA reaching 89.64% mIoU. Our proposed model achieved the highest scores across all metrics, with 90.85% mIoU, 94.77% mPA, 94.62% mRecall, and 94.69% mF1. Compared to the original Segformer, it improved mIoU by 2.60%, mPA by 0.31%, mRecall by 0.44%, and mF1 by 0.28%, confirming the effectiveness of the proposed improvements.

In this experiment, to comprehensively evaluate the performance of our proposed model, we selected four representative images containing at least five of the predefined categories: vegetation, cultivated land, buildings, land, mining area, and background. A total of eight models, including our proposed one, were compared through visual analysis to highlight their segmentation capabilities in UAV-based mine restoration scenes.

The selected images encompassed diverse and challenging scenarios, ensuring a thorough assessment of each model’s ability to handle complex spatial patterns and category differentiation. The predicted outputs of all models were overlaid on the original images, facilitating direct visual comparison across semantic categories. This approach enabled clear observation of each model’s performance in terms of segmentation accuracy, boundary delineation, and feature representation.

**Figure 7:**
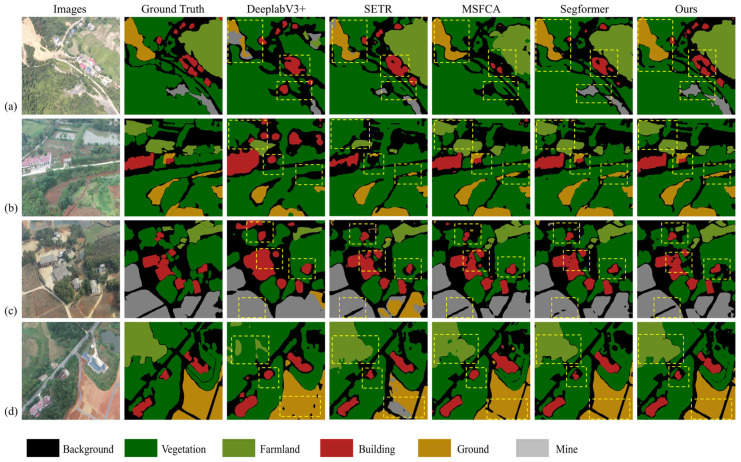
Visualization results of the various methods on the HNMUD.

As illustrated in Figure 7, U-Net and PSPNet exhibited poor recognition performance in complex scenes, with completely incorrect predictions for the central region in Figure 7b. DeeplabV3+ and SETR misclassified several categories, while Swin Transformer and Mask2Former struggled with boundary details and fine-grained structures. Segformer achieved improved performance relative to the aforementioned models; however, it still exhibited inaccuracies in edge detail preservation. MSFCA also showed some ambiguity in detecting small targets. In contrast, our proposed model demonstrated superior accuracy in category recognition and boundary refinement across all tested images.

The visual results clearly indicate that, while existing models achieved acceptable performance in mine restoration scene segmentation, our model delivered more precise results, particularly in distinguishing semantically similar categories, such as vegetation and cultivated land. Furthermore, it maintained consistent accuracy along object boundaries, reducing misclassification and boundary ambiguity. Overall, the proposed model achieved enhanced segmentation precision and exhibited strong generalization capabilities in multi-category UAV image segmentation tasks for mine restoration monitoring.

#### 4.2.2. Experiments on the Aeroscapes Dataset

To evaluate the model’s performance on a public dataset, we conducted experiments on the Aeroscapes UAV remote sensing dataset. The comparison models remained consistent with those used on the HNMUD. As shown in Table 3, the proposed model achieved the best performance across all metrics, with an mIoU of 84.20%, mPA of 91.17%, mRecall of 91.41%, and mF1 of 91.25%. Compared to the original Segformer, the proposed model improved mIoU by 2.74%, mPA by 1.92%, mRecall by 2.05%, and mF1 by 1.93%. In comparison with CNN-based models (U-Net, PSPNet, and DeepLabv3+), the mIoU increased by 13.32%, 8.39%, and 6.96%, respectively, with notable improvements in mPA, mRecall, and mF1. Compared to Transformer-based models (SETR, Swin Transformer, and Mask2Former), the mIoU was higher by 5.61%, 2.84%, and 2.29%, respectively, along with consistent gains in the remaining metrics. The proposed model also outperformed recent models, such as MSFCA and EMR-HRNet, in all evaluation criteria.

**Figure 8:**
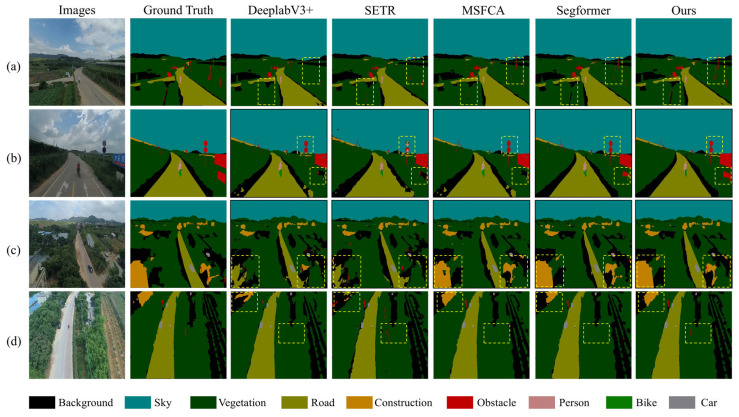
Visualization results of the various methods on the Aeroscapes dataset.

To further evaluate the generalization capability of the proposed model, we selected four representative images for qualitative visualization. These images encompassed various semantic categories, including ‘background’, ‘person’, ‘bike’, ‘car’, ‘obstacle’, ‘construction’, ‘vegetation’, ‘road’, and ‘sky’. The selected samples varied in viewing angles and distances, covering both close-range and far-field scenes, and all contained small objects (e.g., ‘person’, ‘bike’, ‘car’, and ‘obstacle’). Additionally, we present a legend illustrating the semantic labels for each category, as shown in Figure 8. Traditional models, such as U-Net and PSPNet, exhibited suboptimal segmentation performance, with evident category confusion and poor delineation of object boundaries, particularly in densely structured areas. DeeplabV3+ and SETR achieved moderate improvements but still struggled to accurately segment fine-grained structures, such as vehicle contours and building edges. Swin Transformer and Mask2Former produced more refined results; however, noticeable boundary fragmentation persisted in complex regions. Although Segformer performed relatively well, it still suffered from edge blurring and category mixing in occluded or shadowed areas. MSFCA also showed some ambiguity in detecting small targets. In contrast, our proposed model demonstrated superior capability in distinguishing semantic categories and extracting fine-edge details, especially in challenging scenarios.

In contrast, our model consistently achieved superior visual segmentation results, accurately distinguishing between adjacent categories and preserving boundary integrity. It exhibited strong robustness in complex road scenes, effectively segmenting small objects and maintaining contextual consistency across the image. These results validate the generalization strength of our model beyond the proprietary HNMUD, demonstrating its applicability to real-world UAV semantic segmentation tasks across different domains.

### 4.3. Ablation Experiment

To validate the effectiveness of each component in the proposed MSFE-FPN framework, we conducted a series of ablation experiments on the HNMUD. The MSFE-FPN framework is composed of three modules: FPN, SFA-PPM, and ELA. We incrementally integrated these modules into the baseline Segformer-B0 model to evaluate their individual and combined contributions to segmentation performance. As shown in Table 4, introducing the FPN module alone improved the mIoU from 88.25% to 88.58%, and increased mPA, mRecall, and mF1 to 92.52%, 93.54%, and 93.24%, respectively. Adding the proposed SFA-PPM module further boosted performance, achieving 89.82% mIoU and over 94% in all other metrics, demonstrating the module’s effectiveness in selectively aggregating multi-scale contextual features. Finally, incorporating the ELA module into the model with both FPN and SFA-PPM resulted in the best performance, with the mIoU reaching 90.85%, mPA at 94.77%, mRecall at 94.62%, and F1-score at 94.69%.

These results highlight the complementary and synergistic benefits of the three modules, confirming that the complete MSFE-FPN framework significantly enhanced segmentation accuracy and robustness in UAV-based mine restoration scenarios.

To ensure the generalization capability of the model and maintain fairness in comparison, we conducted ablation experiments on the Aeroscapes dataset to evaluate the performance of the proposed framework.

As presented in Table 5, integrating the FPN module into the baseline model improved the mIoU from 81.46% to 82.55%, with corresponding increases in mPA, mRecall, and mF1 to 89.92%, 90.12%, and 90.08%, respectively. Further incorporation of the SFA-PPM module led to significant performance gains, achieving an mIoU of 83.68%, with mPA, mRecall, and mF1 improving to 90.73%, 91.04%, and 90.92%, respectively. Finally, adding the ELA module resulted in the best overall performance, with the mIoU reaching 84.20%, mPA improving to 91.17%, mRecall to 91.41%, and mF1 to 91.25%.

These results underscore the complementary and synergistic contributions of the three modules, validating the proposed framework’s ability to deliver remarkable improvements in segmentation accuracy and robustness for UAV remote sensing applications.

## 5. Conclusions

In this paper, we proposed an improved semantic segmentation framework tailored for UAV-based mine restoration scene understanding, addressing the challenges of complex backgrounds and fine-grained object boundaries. Building upon the lightweight and efficient Segformer architecture, we introduced a multi-scale feature enhancement framework, termed MSFE-FPN, which integrated three key components: FPN for hierarchical feature fusion, SFA-PPM for selective multi-scale context aggregation, and ELA for enhancing local feature discrimination and spatial localization.

To validate the effectiveness of each component, we conducted extensive ablation studies and comparative experiments on a newly constructed private dataset, the HNMUD, and a public benchmark, the Aeroscapes dataset. The results demonstrated that each module contributed positively to segmentation performance, and their combination achieved significant improvements. Our final model achieved an mIoU of 90.85% on the HNMUD, outperforming the original Segformer and other classic baselines. Moreover, experiments on the Aeroscapes dataset further verified the generalization ability of the proposed method.

Overall, our work provided a robust and scalable solution for fine-grained semantic segmentation in UAV-based ecological monitoring tasks. In future work, we aim to explore temporal modeling for multi-temporal UAV sequences and introduce lightweight deployment techniques to enable real-time segmentation in field applications.

## Figures and Tables

**Table 1:** Experimental running software configuration and hardware configuration.

Parameter	Configuration
CPU	Intel Xeon Gold 5218R
GPU	NVIDIA GTX 3090
Memory	128G
Operating System	Windows10
CUDA	Cuda 11.8
Pytorch	Pytorch 2.0.1

**Table 2:** Comparison of results among different models on the HNMUD.

Model	mIoU (%)	mPA (%)	mRecall (%)	mF1 (%)
U-Net [12]	62.43	81.25	73.26	75.90
PSPNet [22]	67.14	83.47	82.28	82.84
Deeplabv3+ [15]	76.20	86.35	85.97	86.13
SETR [18]	76.59	86.69	86.57	86.48
Swin Transformer [23]	83.78	89.98	89.68	89.60
Mask2former [24]	84.32	91.26	91.46	91.36
Segformer [19]	88.25	92.10	93.16	92.83
EMR-HRNet [35]	88.45	92.63	92.47	92.58
MSFCA [36]	89.27	93.85	93.54	93.67
**Ours**	**90.85**	**9** **4.** **77**	**9** **4.6** **2**	**9** **4.** **69**

**Table 3:** Comparison of results among different models on the Aeroscapes dataset.

Model	mIoU (%)	mPA (%)	mRecall (%)	mF1 (%)
U-Net [12]	70.88	81.25	79.26	80.90
PSPnet [22]	75.81	85.11	84.35	84.75
Deeplabv3+ [15]	77.24	86.24	85.52	85.87
SETR [18]	78.59	86.69	86.57	86.48
Swin Transformer [23]	81.36	88.37	87.96	88.12
Mask2former [24]	81.91	89.87	89.19	89.50
Segformer [19]	81.46	89.25	89.36	89.32
EMR-HRNet [35]	82.93	88.31	89.02	88.46
MSFCA [36]	83.27	89.82	90.24	90.07
**Ours**	**84.20**	**91.17**	**91.41**	**91.25**

**Table 4:** Results of ablation tests on the HNMUD (√ indicates the use of the improved module, while - indicates the absence of the improved module).

Baseline	FPN	SFA-PPM	ELA	mIoU (%)	mPA (%)	mRecall (%)	mF1 (%)
√	-	-	-	88.25	92.10	93.16	92.83
√	√	-	-	88.58	92.52	93.54	93.24
√	√	√	-	89.82	94.28	94.58	94.37
√	√	√	√	**90.85**	**9** **4.** **77**	**9** **4.6** **2**	**9** **4.** **69**

**Table 5:** Results of ablation tests on the Aeroscapes dataset (√ indicates the use of the improved module, while - indicates the absence of the improved module).

Baseline	FPN	SFA-PPM	ELA	mIoU (%)	mPA (%)	mRecall (%)	mF1 (%)
√	-	-	-	81.46	89.25	89.36	89.32
√	√	-	-	82.55	89.92	90.12	90.08
√	√	√	-	83.68	90.73	91.04	90.92
√	√	√	√	**84.20**	**91.17**	**91.41**	**91.25**

## Data Availability

The data presented in this study can be obtained upon request from the corresponding author. Other datasets can be obtained from their original papers.
